# Preventive Medicine: Its Achievements, Scope and Possibilities

**Published:** 1904-08

**Authors:** Hermann M. Briggs

**Affiliations:** New York; General Medical Officer, New York City Department of Health; Professor of Therapeutics and Clinical Medicine and Adjunct Professor of Medicine, University and Bellevue Hospital Medical College


### PREVENTIVE MEDICINE: ITS ACHIEVEMENTS, SCOPE AND POSSIBILITIES.*

By HERMANN M. BIGGS, M.D., New York;

General Medical Officer, New York City Department of Health; Professor of Therapeutics and Clinical Medicine and Adjunct Professor of Medicine, University and Bellevue Hospital Medical College.

The terms sanitary science, public or State medicine, and preventive medicine, have frequently been used as almost synonymous. The latter term, "preventive medicine," however, has sometimes been restricted in its application to the prevention of the infectious diseases. In the broad sense, preventive medicine comprises both general prophylaxis and individual prophylaxis, and applies to all forms of disease, not simply to the infectious disease. Preventive medicine is an applied science, which deals with the preservation of the health both of the individual and of the community.

No subject more vitally concerns the welfare of a community than that pertaining to its healthfulness. How the inhabitants live and how and at what age they die, what is the extent and character of the morbidity occurring among them and what are its causes, are questions of momentous importance. They are essential features in the problem, whose solution will teach men how they may live longer, healthier, and therefore happier lives.

In rural districts the average healthfulness of the inhabitants depends largely on natural conditions, such as elevation, climate, soil, and, to a much less extent, on the artificial conditions produced by the handiwork of man. In densely populated cities, on the contrary, the natural conditions become relatively unimportant features in determining the degree of healthfulness, so much do they become subordinated in many cases to the artificial conditions which have resulted from the lives, labor and occupations of the inhabitants. It may be said with certain limitations that the inhabitants of any city in the temperate zone now have it largely in their power to determine what degree of healthfulness their city

*Abstract of an Address on State Medicine at the Fifty-fifth Annual Session of the American Medical Association at Atlantic City, June 7 to 10, 1904. Courtesy of the Journal of the American Medical Association.

shall have. A high morbidity and a high mortality in any urban population depend largely on some remediable features of an unsanitary character, such as the sewerage of the city, the occupations, habitations, food and water supplies, and the habits of the inhabitants—all factors which lie to a great extent within their control. Hence, when any city in modern times has a high annual death-rate, for example, 30 to 35 per 1,000, as was formerly the case in New York, Liverpool and other cities, instead of a death-rate of 17 to 18 or 19 per 1,000, as now exists in London, Berlin and New York, it is because conditions are permitted to exist partly from the neglect, indifference and ignorance of the people, and partly from the lack of proper facilities and necessary efficiency on the part of the authorities.

The expectation of life at birth in certain manufacturing cities of England has been but little more than one-half that of the healthiest districts of England. Until recent years it was twenty-five or twenty-six years in Liverpool and Manchester, thirty-seven years in London and forty-six years in Surrey, a healthy country district. Even at the age of thirty years, the expectation of life was seven years more for England as a whole than it was for the inhabitants of Manchester. I have estimated that the expectation of life at birth in New York City in 1866 was only a little more than twenty-five years, while in 1903, calculated on the death-rate for that year, it had almost doubled, and equaled about forty-two years. For the seven-year period ending in 1873 the death-rate under five years in New York City was 123 per 1,000 of the population at these ages. For the year 1903 the death-rate under five years was 56. On the present estimated population at these ages, this reduction equals a saving of more than 28,000 lives annually.

In the seventeenth and eighteenth centuries the average annual death-rate throughout the civilized world was at least 50 per 1,000 of the population, and probably it was much more than this. From 1628 to 1635 in London, these years being free from pestilence, the average death-rate was 50 per 1,000, and the absolute annual mortality for twenty-four years, from 1620 to 1643, was over 70 per 1,000. The death-rate during 1902 in London was only 17 and a fraction per 1,000. The mean expectation of life in London for the decennial period, 1771-1780, as calculated by Price's table, was 19.6 years, the annual mortality being 51 or 52 per 1,000; while from 1831 to 1835, although an epidemic year is included, the death-rate had decreased to about 32 per 1,000. In 1894 the mean expectation of life at birth for all England had more than doubled that of 1780 in London, so that it was nearly forty-one years, and for London alone it was thirty-seven years. From 1771 to 1780 in London not less than five in each thousand of the population died annually of smallpox; in 1810 this mortality had sunk to two per 1,000, and in 1835 to 0.8, and in recent years it has been an absolutely insignificant decimal. Fever as a cause of death declined in almost the same ratio, from 6.21 in the decimal period ending in 1780 to 2.64 in 1810, and 1.14 in 1835. In the sixteenth century fever, plague, cholera and dysentery destroyed annually 31 out of every thousand of the population of London, or nearly twice the total deaths occurring now from all causes. These diseases at the present time, as causes of death, do not equal in London 2 per 1,000 annually of the population.

In old New York City (comprising the boroughs of Manhattan and the Bronx), for the seven-year period ending in 1873, 616 out of every thousand children born died during the first five years of life. As computed by the death-rate under five in 1903, 28 would have died during these same five years. This mortality while still far too high, equals a reduction of nearly 60 per cent., and as calculated for the estimated population at these ages, means a saving of about 20,000 lives a year in these boroughs. The death-rate in the period over five years of age has naturally shown a much smaller decrease, although it has fallen from 19 or 20; as it was in 1865 and 1866, at the time of the organization of the Department of Health of New York City as at present constituted, to about 14 for the year 1903, being a reduction of nearly one-third. This has been accomplished notwithstanding the fact that the old city has increased during this time from a population of about 750,000 to more than 2,200,000.

It does not seem necessary or desirable for our present purpose to consider in detail the causes of this decrease or the special diseases in which it has taken place, but the facts suffice to indicate the great achivements of preventive medicine during the past four decades.

The scope of the sanitary measures in force with reference to the ordinary infectious diseases has in recent years been greatly broadened. More than ten years ago the Board of Health in New York assumed the position that all facilities and procedures looking to the prevention and cure of the infectious diseases should be afforded by the Department of Health. The establishment of the methods for bacteriological diagnosis and the facilities for the serum treatment had their origin there, and have since been widely followed.

General prophylaxis, or that proportion of preventive medicine which should come directly under the supervision of the health authorities, has or should have, a broader scope than that indicated, and should include the supervision, not only of those diseases which have generally come within sanitary surveillance, but also all the infectious diseases which from their nature are to a greater or less extent preventable, in addition to all other forms of disease which are the result of unsanitary living, occupations, habitations or surroundings, including those diseases arising from manufacturing, mining, or other industries which, on account of their nature, are peculiarly or especially injurious to health. It should also include with greatly increased powers and breadth of jurisdiction the surveillance of the water and food supplies and the sewage disposal.

There has long existed a belief in the medical profession, and to a considerable extent it has also been a part of the conception of the sanitary authorities of their functions, that the registration of a case of any kind of infectious disease involved some surveillance of such case by the authorities. The slightest consideration would at once show that with relation to many of the diseases mentioned no such surveillance could be of any real service, and it might be a source of annoyance to the sick person, the family and the attending physician. Much good, however, would result, in my opinion, by such a broadening of the scope of sanitary work, without causing annoyance or being detrimental to any one. I should like, however, to point out that more than a year ago the Board of Health of New York City in the revision of its sanitary code included a number of new sections in relation to the infectious diseases, covering to a large extent the diseases above mentioned. These sections, with the accompanying notes, read as follows:

INFECTIOUS DISEASES.

SEC. 133. It shall be the duty of every physician to report to the Department of Health, in writing, the full name, age and address of every person suffering from any one of the infectious diseases included in the list appended, with the name of the disease, within twenty-four hours of the time when the case is first seen:

A. *Contagious* (very readily communicable): Measles, rubella (Rötheln), scarlet fever, variola (smallpox), varicella (chicken-pox), typhus fever, relapsing fever.

B. *Communicable*: Diphtheria (croup), typhoid fever, Asiatic cholera, tuberculosis (of any organ), plague, tetanus, anthrax, glanders, epidemic cerebro-spinal meningitis, leprosy, infectious diseases of the eye (trachoma, suppurative conjunctivitis), puerperal septicemia, erysipelas, whooping cough.

C. *Indirectly Communicable* (through an intermediary host): Yellow fever, malarial fever.

SEC. 134. It shall be the duty of the commissioners or managers, or the principal, superintendent or physician, of each and every public institution or dispensary in this city, to report to the Department of Health, in writing, the full name, age and address of any person suffering from any one of the infectious diseases included in the list appended, with the name of the disease, within twenty-four hours of the time when the case is first seen:

A. *Communicable:* Influenza, lobar pneumonia, bronchopneumonia, infectious diseases of the gastrointestinal canal (dysentery, cholera morbus, cholera infantum, summer diarrheas of infants).

B. *Parasitic Diseases of the Skin*: Scabies, *Tinea tonsurans* impetigo (contagious), favus.

It will be noted, first, that two main groups of diseases are specified. In the first group, including the more readily communicable diseases, and some others which are not so regarded, registration is compulsory in all cases coming under the observation either of a private attending physician or an institution. These are separated for convenience under three heads: (*a*) The contagious or very readily communicable; (*b*) the communicable, including diphtheria, typhoid fever, Asiatic cholera, tuberculosis, and a number of others, and (*c*) the indirectly communicable, including only two diseases—yellow fever and malarial fever—which are transmitted solely through an intermediary host. In these three classes compulsory registration is required because in all of the diseases in these classes preventive measures, properly executed, are certainly efficient to a very large extent. As to the propriety of the compulsory registration of all the diseases included under the first heading, "The contagious or very readily communicable diseases," I assume that there can be no reasonable difference of opinion. Measles, Rötheln, scarlet fever, varicella, variola, typhus fever and relapsing fever, certainly without question, should be reported to the sanitary authorities. The necessity for reporting Rötheln and varicella rests more in the danger of confusion of these diseases with measles and varioloid, respectively, than because of the importance of the diseases themselves. Our experience in New York has shown again and again that outbreaks of smallpox have resulted from the failure of inexperienced physicians to recognize mild cases, these having been mistaken for varicella and treated as such. Every case of varicella occurring in the city must, therefore, be reported, and all cases occurring in adults are immediately seen by a trained diagnostician. I say all cases of varicella in adults, because experience has shown that very frequently varicella in an adult is a mild case of variola.

In the second class, called simply "the communicable diseases," there might be some question as to the propriety of the reports in some instances. There would be none, however, with regard to diphtheria, typhoid fever, Asiatic cholera, plague, tetanus, anthrax, glanders and leprosy.

There can not now be, nor can I conceive that there ever will be again, any serious question as to the necessity or propriety for the registration and sanitary surveillance of tuberculosis, although this has been a subject of very bitter dispute and although this disease has come actually under the close supervision of the sanitary authorities in only three or four cities in the United States, and of practically none in Great Britain or on the Continent. Pulmonary tuberculosis was included in the list of communicable and reportable diseases by the New York City Board of Health ten years ago, and the measures adopted for its surveillance have been gradually extended and the lines for its restriction have been drawn closer and closer as each year has passed. Some doubt, however, might properly arise as to the necessity of the inclusion of other forms of tuberculosis than pulmonary tuberculosis in the registration returns, because for the most part these other tubercular diseases are not communicable, or very slightly so.

The infectious diseases of the eye—trachoma and suppurative conjunctivitis—should be included because of the importance of the adoption of preventive measures in the public schools. Few persons had any conception of the great prevalence of these diseases of the eye in the public schools of New York until after the Department of Health adopted some comprehensive measures for their surveillance. Such a vast number of cases was found that the Board of Health was forced to establish special dispensaries and provide special hospital facilities for their care. Only within a few weeks a second hospital and dispensary has been opened, and in the original one in connection with Gouverneur Hospital an average of about 500 patients a day were treated during the year 1903, and nearly 5,000 cases were operated on for trachoma.

Puerperal septicemia should be included in the registration returns because modern aseptic midwifery has shown that in most instances this is a strictly preventable disease, and excepting rare cases of difficult or prolonged labor, its occurrence may be regarded as the result of uncleanliness, negligence or incompetence.

No one, I fancy, would seriously question the propriety of including erysipelas and whooping cough in the registration returns. So far as this country is concerned I assume that typhus fever, Asiatic cholera, plague, leprosy and yellow fever are diseases which will never again call for anything more than occasional or emergency measures for the disposal of isolated cases or small groups of cases.

The registration of cases of malarial fever should be required, not because close surveillance of individual cases is necessary, but because the sanitary authorities should know where the sites of infection are, where the breeding-places of the *Anopheles* mosquitoes are to be found, in order that they may adopt proper means for determining the cause of such areas of infection and the proper measures for their removal. In certain sections of New York City investigation has shown that in some instances almost every person living within certain small areas has suffered from malarial fever during the spring and summer months of a single season. Such outbreaks are without any question the result of local remediable unsanitary conditions, which furnish the breeding-places for the mosquitoes. But without special information as to the existence of the case, only to be obtained through the registration of malarial fever, knowledge of the presence of such unsanitary conditions and their removal may become difficult or impossible. The surveillance in their homes of the individual cases reported is, of course, not contemplated. That conditions similar to those found in New York exist in other communities, I have no doubt.

Section 134 of the Sanitary Code of New York, covering the second group, deals with infectious diseases of a somewhat different type, and in these the Sanitary Code requires the reporting to the department by public institutions of all cases coming under their observation; but it only requests physicians to report them and does not require such reporting.

Class (*a*) in this group includes some of the more important acute infectious diseases of the upper respiratory tract, namely, influenza, lobar pneumonia and bronchopneumonia, and certain of the more common acute infectious diseases of the gastrointestinal canal, namely, dysentery, cholera morbus, cholera infantum and the summer diarrheas of infants.

The acute diseases of the respiratory tract referred to should, in my opinion, be registered, because they are infectious, because they are of prime importance as causes of death, and in order that general and special investigation may be instituted for the collection of data bearing on the modes of infection and the sources and causes of their prevalence and their distribution. New York City has during the last winter experienced the greatest prevalence of these diseases which it has had for many years. In the first four months of the year more than 6,000 deaths occurred from influenza, lobar pneumonia, bronchopneumonia and acute bronchitis.

Investigation in New York and other localities have shown that serious outbreaks of diarrhea and dysentery have occurred in various institutions and in local areas which were probably, if not certainly, preventable, but concerning which the sanitary authorities have had no information. It seems evident, therefore, that these diseases should be made notifiable.

Notification of the parasitic diseases of the skin should be required chiefly because of the liability to the extension of these diseases among children in the public schools.

There are certain very large and important questions of prophylaxis connected with the prevalence of typhoid fever and the milk and water supply of our large communities. The occurrence of great outbreaks of typhoid fever, such as the epidemics at Ithaca, N. Y., and Butler, Pa., during the last year, the prevalence in Philadelphia, Pittsburg and some other cities of the United States and the extensive distribution in the rural districts, constitute a disgrace to American preventive medicine and should arouse the sanitary authorites, the medical profession and the people to the provision of efficient measures and the enactment of suitable legislation for its prevention.

The annual cost of typhoid fever in sickness and death throughout the United States would be computed in terms of tens of millions of dollars. All of this loss we know is certainly preventable. If only a small portion of the annual outlay made by the country for sickness and death from this cause were expended in the education of the people as to the cause and the methods of prevention of this disease and the provision of proper water and sewerage system, typhoid fever might soon become a rare affection. Unfortunately the city sanitary authorities are often almost powerless in this matter, as their jurisdiction does not extend over the watersheds which supply with water the people under their care, and a supervision by them of the sources of the milk supply is also frequently absolutely impossible.

In New York City detailed investigations of all the cases of typhoid fever occurring within the city limits were instituted several years ago and have been continuously carried on. These show that at least twenty-five per cent. of the cases occurring in the city have *certainly* received their infection outside of New York City, and a considerable percentage more have *probably* received it outside. Five or six per cent. are the result of contact with other cases, and the remainder are in all probability almost wholly the result of infection through milk or oysters.

A serious objection which may be urged against the adoption of the measures here recommended in regard to the registration of many infectious diseases is the increased labor thrown on physicians which their enforcement would involve. The question may properly be asked: Whether the sanitary authorities in the interest of the general public may call on the medical profession for the expenditure of so much time and labor without making any compensation in return. There are thirty-five diseases included in the New York list. The English authorities pay a fee for the notification of each case of infectious disease, and I believe that this course might properly be pursued in this country; but this has not been the custom here, and the authorities generally are not now provided with the funds necessary for this purpose.

When the department of Health of New York City established its bacteriological laboratories in 1892, measures were adopted to make some return to physicians for their interest and labor in connection with the sanitary surveillance of the infectious diseases. A systematic plan was then inaugurated, and has since been consistently followed, to secure their cooperation by offering free bacteriological examination in the diagnosis and surveillance of various forms of infectious disease. This was first applied to Asiatic cholera in 1892, during the outbreak in New York harbor, and has since been extended to all of the diseases in which existing scientific knowledge made it possible, cerebrospinal meningitis being the latest disease added to this list. The establishment of this system of free bacteriological examinations in the diagnosis and surveillance of the infectious diseases, and the provision of facilities throughout the city for the collection of specimens and the sending of reports to physicians, constituted an entirely new departure in sanitary work and has been of the greatest value. The example of New York has been followed by the sanitary authorities in most of the large cities of this country, by many State authorities, and by authorities generally, in Great Britain and to a certain extent on the Continent. These examinations constitute the return which the authorities here have made to physicians for the reporting of cases and for their assistance and cooperation in their care. This return has a very considerable money value, when we remember that for such examinations the usual fee is $5, and as physicians can not, as a rule, make such examinations themselves, they must work without the assistance thus afforded, unless they appeal to the regularly constituted laboratories.

What now are the possibilities of preventive medicine as concerns the larger centers of population? In answering this question we may first take as the basis of our reply the conditions found in some of the large cities with the lowest death-rates, or still better, the conditions found in some healthy rural districts. In considering the crude death-rates, as ordinarily reported, however, one must not ignore the influence of the sex and age distribution of the population on the death-rate. As is well-known, the death-rates in the period of life between five and forty-five years of age are very low, and the death-rate among women is lower than among men. If, then, in a city there is a relative excess of the population in the middle periods of life, or an unusual preponderance of women, we may have a crude death-rate which seems very low, but which when corrected may be relatively high. For example, the death-rate of New York City, as corrected by the English life tables, would probably be at least three points higher than it is. For 1894 the gross rate was 22.76, and the rate corrected by the English table was 26.46. The corrected death-rate of some of the large, rapidly growing Western cities would probably be five or six points higher than the crude death-rate reported. The death-rate of London at present averages about 17 per 1,000, and when corrected about 18.5 per 1,000, while the Berlin death-rate is somewhat lower than this. The crude death-rate of Surrey, a healthy rural district in England, is 14 and a fraction, and when corrected would probably be 15.

We can hardly use for a basis of comparison the conditions in any of the cities of this country with very low rates, because the rates have not been corrected, and the basis on which they are calculated can not always be accepted as correct. Taking all the facts together, and judging of the duration of human life as we know it, we may accept a corrected death-rate of 14 or 15 in a large city as a goal toward which preventive medicine should strive and hope to achieve. If we consider the question from another point of view, as to what is possible in the reduction of the death-rate from distinctly preventable diseases, we would reach a conclusion not unlike the previous one.

In New York City it may be assumed that the present tuberculous death-rate of 2.7 may, under proper surveilllance, with certainty be reduced to 1.2, and 1.5 points would thus be subtracted from the crude rate. The diphtheria death-rate, already reduced 65 per cent. by the use of antitoxic serum, should certainly be reduced to one-third of what it is at present; it is now about .6 per 1,000 population. Similar reductions should be possible in the death-rate from typhoid fever, measles and scarlet fever. The diarrheal diseases of infants have already been reduced in the last twelve years to one-half of their former prevalence, and should still be reduced more than 50 per cent. Thus more than three points may be subtracted from the crude rate in New York, which in 1903 was 18.18. The rate would then be 15+. The rate for 1903 was the lowest in the history of the city.

The most fatal affections with which we have now to deal in New York are the acute respiratory diseases, including influenza, lobar pneumonia, bronchopneumonia and acute bronchitis. The average death from this group of diseases now exceeds three per 1,000 of the population, and in years when there is an epidemic prevalence of these diseases the death rate may be four or more, as seems likely to be the case this year.

The advances in preventive medicine have as yet not only had no influence in reducing the death-rate from this group of diseases, but, on the contrary, there has been a slow and continuous increase. I can not at this moment point out the method or the means by which a reduction is to be brought about, but still I firmly believe that with fuller knowledge such a reduction will become possible in the great cities where these diseases are so prevalent.

Something can be accomplished through the closer sanitary surveillance of the occupations and conditions under which the lower classes work, especially the noxious trades. Something, too, may be done through a closer supervision of foods to ensure their purity and freedom from adulteration.

A very difficult problem arises in connection with the diseases of the circulatory apparatus and the kidneys. My investigations have shown that in New York City a large increase has taken place, due to these causes, during the last twenty years. The acute respiratory diseases, cancer and diseases of the circulatory apparatus and the kidneys are the only important causes of death which have shown an increase during this period. The increase in cancer amounts to about 15 per cent., and the increase in the acute respiratory diseases amounts to about 15 per cent., while the increase in the diseases of the circulatory apparatus and kidneys combined equals about 40 per cent. In making this statement I have taken fully into consideration the possible influence of greater accuracy in the death returns, *i. e.*, the inclusion formerly of these under other causes of death ; but after making all allowances, it seems to me without question that an increase equaling 40 per cent. has taken place.

